# ScType enables fast and accurate cell type identification from spatial transcriptomics data

**DOI:** 10.1093/bioinformatics/btae426

**Published:** 2024-06-27

**Authors:** Kristen Nader, Misra Tasci, Aleksandr Ianevski, Andrew Erickson, Emmy W Verschuren, Tero Aittokallio, Mitro Miihkinen

**Affiliations:** Institute for Molecular Medicine Finland (FIMM), HiLIFE, University of Helsinki, Helsinki 00290, Finland; iCAN Digital Precision Cancer Medicine Flagship, University of Helsinki and Helsinki University Hospital, Helsinki 00290, Finland; School of Medicine, Koç University, Istanbul 34450, Turkey; Institute for Molecular Medicine Finland (FIMM), HiLIFE, University of Helsinki, Helsinki 00290, Finland; iCAN Digital Precision Cancer Medicine Flagship, University of Helsinki and Helsinki University Hospital, Helsinki 00290, Finland; iCAN Digital Precision Cancer Medicine Flagship, University of Helsinki and Helsinki University Hospital, Helsinki 00290, Finland; Research Program in Systems Oncology, University of Helsinki, Helsinki 00290, Finland; Institute for Molecular Medicine Finland (FIMM), HiLIFE, University of Helsinki, Helsinki 00290, Finland; Institute for Molecular Medicine Finland (FIMM), HiLIFE, University of Helsinki, Helsinki 00290, Finland; iCAN Digital Precision Cancer Medicine Flagship, University of Helsinki and Helsinki University Hospital, Helsinki 00290, Finland; Institute for Cancer Research, Department of Cancer Genetics, Oslo University Hospital, Oslo 0310, Norway; Oslo Centre for Biostatistics and Epidemiology (OCBE), Faculty of Medicine, University of Oslo, Oslo 0372, Norway; Institute for Molecular Medicine Finland (FIMM), HiLIFE, University of Helsinki, Helsinki 00290, Finland; iCAN Digital Precision Cancer Medicine Flagship, University of Helsinki and Helsinki University Hospital, Helsinki 00290, Finland

## Abstract

**Summary:**

The limited resolution of spatial transcriptomics (ST) assays in the past has led to the development of cell type annotation methods that separate the convolved signal based on available external atlas data. In light of the rapidly increasing resolution of the ST assay technologies, we made available and investigated the performance of a deconvolution-free marker-based cell annotation method called scType. In contrast to existing methods, the spatial application of scType does not require computationally strenuous deconvolution, nor large single-cell reference atlases. We show that scType enables ultra-fast and accurate identification of abundant cell types from ST data, especially when a large enough panel of genes is detected. Examples of such assays are Visium and Slide-seq, which currently offer the best trade-off between high resolution and number of genes detected by the assay for cell type annotation.

**Availability and implementation:**

scType source R and python codes for spatial data are openly available in GitHub (https://github.com/kris-nader/sp-type or https://github.com/kris-nader/sc-type-py). Step-by-step tutorials for R and python spatial data analysis can be found in https://github.com/kris-nader/sp-type and https://github.com/kris-nader/sc-type-py/blob/main/spatial_tutorial.md, respectively.

## 1 Introduction

The significance of tissue spatial organization for biological functions has spurred multiple technological advances that permit the analysis of gene regulation directly from tissue locations (Marx 2021). Integral to these developments, spatial transcriptomics (ST) has emerged among the most widely applied methodologies to date. ST serves as a collective term encompassing an array of methodologies that permit spatial profiling of fresh frozen or formalin-fixed paraffin embedded (FFPE) tissue samples. Currently, the most advanced and widely used ST methodologies include 10× Genomics Visium and Xenium, Slide-seq, and GeoMx Digital Spatial Profiler (DSP), which differ in their underlying sampling principles, mRNA detection workflows and expression analysis technologies (Ståhl *et al.* 2016, Rodriques *et al.* 2019, Merritt *et al.* 2020, Moses and Pachter 2022).

Regardless of the ST methodology, an initial step in any spatial expression analysis workflow is to define the cell types within the 2D tissue sample. To this end, several computational methods, mainly employing reference-based deconvolution, have been developed (Elosua-Bayes *et al.* 2021, Cable *et al.* 2022). However, deconvolution algorithms are computationally intensive and require an external single-cell atlas for reference. Given that the ST assays are currently reaching single-cell resolution, we aimed to evaluate the performance of existing, computationally faster algorithms to annotate cell types from ST samples.

In this Application Note, we benchmarked the performance of scType against more classical, but computationally demanding cell-type annotation methods currently used in ST analyses. We originally developed scType as an ultra-fast tool for cell type identification through specific marker combinations from scRNA-seq data (Ianevski *et al.* 2022). Here, we show that scType can accurately annotate prevalent cell types directly from ST data in which a large enough panel of genes is detected, without the need for external single-cell reference data. Furthermore, we illustrate the potential of scType to enhance pathology annotations by offering cell type identifications that are comparable, if not superior, to conventional pathology examinations.

## 2 Software description

ScType analyzes the detected gene signature at each ST spot against a comprehensive marker gene database (scTypeDB), which contains both negative and positive marker genes for various cell types and assigns the most appropriate annotation for the given ST data. ScType annotation is based on a scoring algorithm, in which the specificity of markers across different spots and cell types is ensured within a sample (Ianevski *et al.* 2022). Notably, the scoring considers both positive and negative markers, significantly enhancing accuracy, particularly for rarer cell types. Users can utilize the scTypeDB by default or incorporate their custom marker genes, which is beneficial in specific cases, such as with less-studied model organisms, cell types, or in detailed analyses of highly specialized mouse or human tissues.

To briefly describe the scoring algorithm, the input ST expression matrix is transformed by multiplying it with the cell type specificity scores, resulting in a weighted matrix. This, combined with the set of positive and negative markers, generates positive and negative marker set enrichment matrices. Enrichment matrices are then integrated to derive the cell type enrichment matrix. ScType identifies the cell type by selecting the maximum score across all potential cell types, thus determining the predominant cell type signature for each spot. ScType is open source, available as R and Python implementations (see GitHub for user instructions on spatial data analysis), and also accessible via a web server (Ianevski *et al.* 2022).

## 3 Results

**Figure 1.**
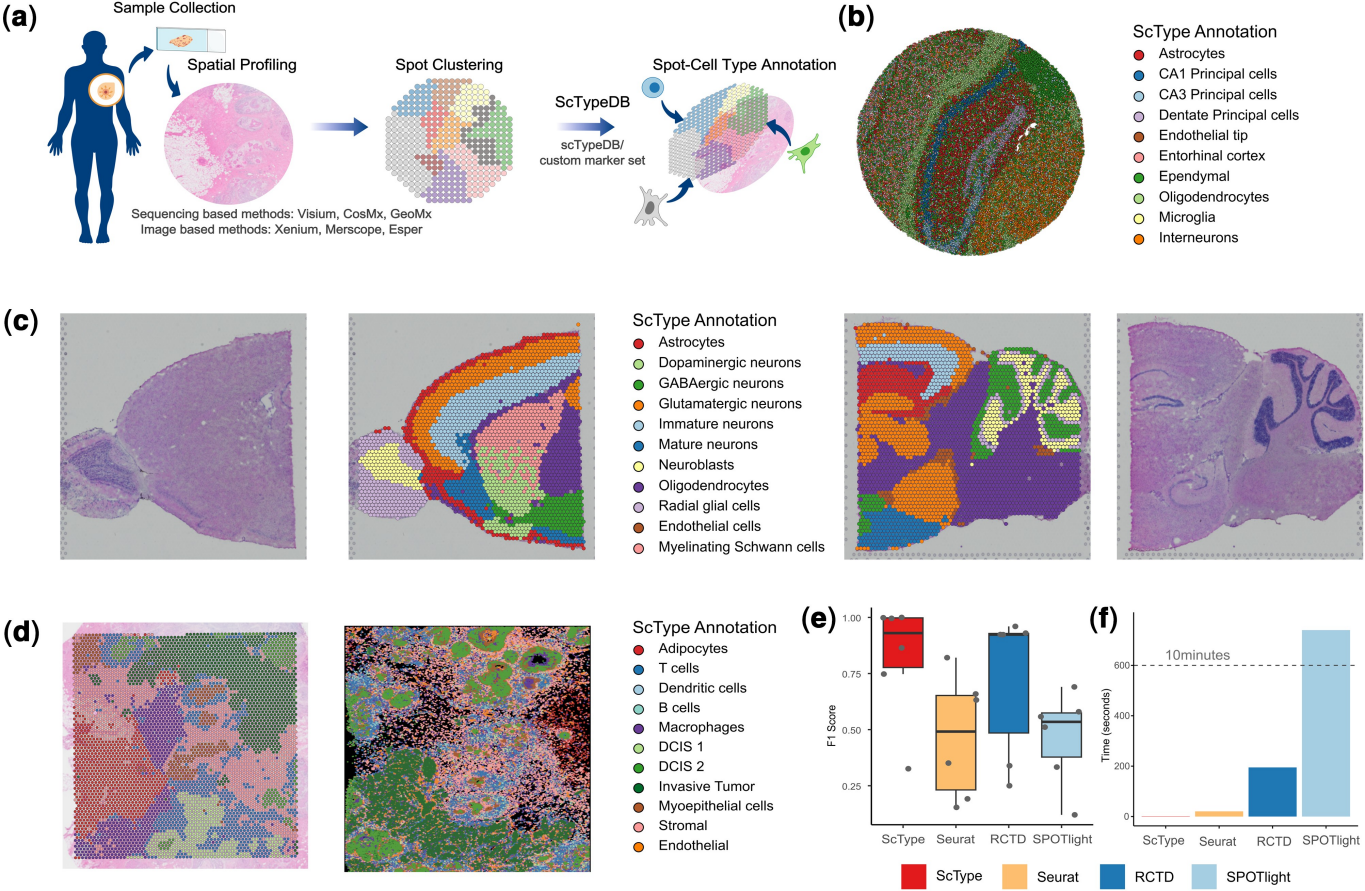
Performance of scType across various tissue samples and spatial technologies. (a) Schematic representation of spatial transcriptomics spot annotation using scType for a human breast tissue sample. The user can either rely on markers in the comprehensive scTypeDB or use their own specific cell type markers. Created with BioRender.com (b) Cell type identification with scType from Slide-seq data of mouse hippocampus. (c) Cell type identification from 10× Visium data using scType with examples of sagittal anterior (left) and posterior (right) mouse brain. (d) Cell type identification with scType from breast cancer Visium (left) and Xenium (right) datasets using pathologist annotations as ground-truth. (e) Comparison of different annotation tools using the F1 score when applied to breast cancer dataset. To correct potential bias resulting from uneven taxonomical depth, the cell type labels were unified according to Supplementary Table S2. Points indicate the different cell types [DCIS #1, DCIS #2, Immune, Invasive, Myoepithelial, Stromal/Endothelial], and horizontal lines indicate the median F1 score. Supplementary Figure S3 shows the results separately in different cell types and also reports precision and recall metrics. Seurat refers to using FindTransferAnchors and TransferData functions in the Seurat version 5.0.1. Individual data points represent predictions of each cell type from the custom marker set. (f) Runtime metrics of different annotation methods when applied to the breast cancer dataset. See Supplementary Table S1 for the spatial dataset characteristics.

In light of the advancements in spatial omics resolution, we explored the possibility of annotating cell types from ST data using deconvolution-independent, marker-based approaches like scType. To ensure robust testing, we compiled spatial omics datasets from different laboratories and sources (Supplementary Table S1). This is important, as different spatial omics methods detect varied amounts of genes, which might affect the cell type classification when using marker-based methods. More specifically, we assessed the performance of scType in both unbiased (Visium, Slide-seq) and targeted (Xenium) datasets (Fig. 1a; Supplementary Table S1).

We initially annotated Slide-seq data from a sagittal tissue section of a mouse hippocampus, where the anatomical locations of different neuronal cell populations are well understood and can be used as ground-truth reference (Stickels *et al.* 2021), guided by mouse brain marker genes identified in (Saunders *et al.* 2018). Strikingly, scType was able to clearly separate different hippocampal anatomical regions with a high degree of detail extremely difficult to achieve with conventional histological methods (Fig. 1b). This fine resolution was evident in various tissue areas such as different regions of the pyramidal layer and dentate gyrus, all without using external reference cell atlas nor computational deconvolution. In addition, scType effectively assigned tissue locations with a cell type label that accurately reflects the function of each hippocampal region (Fig. 1b). For instance, scType correctly placed astrocytes around the stratum variatum, dentate principal cells in the dentate gyrus, and CA1 or CA3 pyramidal neurons into different parts of the pyramidal cell layer (Fig. 1b).

Further testing on the Visium platform, using publicly available sagittal mouse brain data (Visium v1 chemistry), demonstrated that scType, with either default or targeted marker genes, could distinctly separate brain areas in both anterior and posterior sections (Fig. 1c). This separation, unachievable through H&E staining alone, suggests that molecular-based cell type labeling from spatial omics data can facilitate biological discovery by categorizing anatomical tissue areas based on their molecular features (Fig. 1c). More specifically, cell type annotations with scType led to a clear separation of brain structures such as the olfactory bulb, fiber tracks, corpus callosum and different layers of the cortex with indications of abundant cell type labels for each (Fig. 1c).

Interestingly, despite Visium’s lower resolution compared to Slide-seq (resolution: 100 μm versus 10 μm, Supplementary Table S1), scType successfully assigned neuronal cell types to their accurate anatomical locations, such as GABAergic neurons in the cerebellar cortex (Kelsom and Lu 2013), and oligodendrocytes in the corpus callosum (Meyer *et al.* 2018). ScType also captured signatures of Neuroblast-derived cell types in the olfactory bulb granule layer and cerebellar cortex fiber tracks, which it labeled broadly as Neuroblasts, indicating highly convolved signal in these tissue areas (Fig. 1c) (Whitman and Greer 2009). These findings indicate that scType can identify precise anatomical regions and accurately assign major cell types in ST data, even in the absence of reference cell atlases (Fig. 1b and c). To investigate the performance of scType within a disease context, we next annotated spatial areas from a breast cancer spatial omics dataset (Supplementary Table S1). The dataset consisted of matched single cell reference, Visium and Xenium ST samples, as well as pathologist annotations, used as ground-truth, dividing tissue areas into categories, such as ductal carcinoma in situ (DCIS), invasive tumor, immune infiltrations or stromal and adipose tissue (Janesick *et al.* 2023). Interestingly, scType was able to confidently annotate cell types in the Visium data (Fig. 1d), but it failed to do so with Xenium data, indicating that the number of genes profiled by this platform (313 genes) is too low or does not match with the used markers in scTypeDB (Fig. 1d). On the contrary, and as an example of the Visium analyses, areas marked as “Stromal,” “Stromal/Endothelial,” or “Stromal/Endothelial/Immune” by a pathologist were all labeled as “Stromal” by scType, indicating that stromal cells are the most abundant cell type in these locations (Fig. 1d, Supplementary Fig. S1A).

To quantify the annotation accuracy, we followed the reasoning where we considered the predicted cell type labels correct, if they matched the pathology annotation at the same or deeper cell type taxonomy level. For instance, if a spot was labeled as “Immune cell” by the pathologist, cell type assignments of “T cell,” “Dendritic cell,” or “B cell” would all be correct, but “Stromal” would be incorrect (Supplementary Fig. S1A and B). Using such a benchmarking in the breast cancer dataset, we quantified both the accuracy and runtime of two deconvolution-based methods (RCTD and SPOTlight) (Elosua-Bayes *et al.* 2021, Cable *et al.* 2022) and two marker-based methods (scType and Seurat) (Ianevski *et al.* 2022, Hao *et al.* 2023). Notably, scType was able to reach a median F1 score of 0.93 (best deconvolution method was RCTD with median F1 = 0.92), while demonstrating a superior computational speed (run time 1 s versus 3.2 min of RCTD).

To further benchmark the accuracy of the methods to annotate each cell type separately, we employed a simulation approach, where we virtually placed cells of different cell types onto a 2D grid, and probed their expression with 10 μm spatial transcriptomics spots overlaid with the 2D grid. In the simulated data, the methods displayed a comparable performance, which depended on the cell type (Supplementary Fig. S2, Supplementary Table S3). This cell type-specific behavior is in agreement with the real data results, where scType showed improved performance across the cell types, when compared to the other methods (Supplementary Fig. S3). Furthermore, the ultra-fast running time of scType and applicability to high-resolution data makes cell type annotations easy to do and repeat by the users. Another practical benefit of scType is that it does not require any single-cell reference data as its input.

## 4 Conclusions

In this Application Note, we repurposed the existing scType tool to annotate cell types from spatial transcriptomics data and benchmarked its performance against existing spatial cell type annotation tools that utilize various frameworks, both deconvolution and marker-based methods. Importantly, scType was able to accurately identify abundant cell types from datasets generated using Visium or Slide-seq, but lacked similar accuracy in Xenium data, due to a lower number of genes in this assay (313 of genes profiled). The reduced number of genes and the inability to freely choose the probes used by the Xenium assay lead to a significantly reduced scType expression matrix, impacting the performance of scType on Xenium-profiled tissues. Although the number of probes used by Xenium is limited, compared to other assay types, the performance of marker-based cell typing would vastly increase if the whole 313 genes could be designed to fully match the target tissue.

For better understanding of different cell typing methods, we established a pipeline to generate simulated spatial transcriptomics data, in addition to comparing the methods’ performance using data from real tissue. Interestingly, in both simulated and real data, we saw variability in the performance to annotate cell types which was, to some degree, cell type dependent. When using a marker set for cancerous (DCIS) tissue, immune cells such as T-cells were overall the hardest to annotate and cancer cells the easiest, especially with scType. Although users can freely use the default marker sets available through scTypeDB, for more specific tissue types or less-studied model organisms, we encourage users to use their own marker panels. For this, the GitHub R tutorial shows how to create a marker set from reference data, but we note that the users can organize their marker sets using any way they feel appropriate.

As the gene signatures and cellular resolution of the ST assays continue to expand, currently approaching single-cell resolution and >30 000 genes, the applicability and accuracy of deconvolution-free and marker-based methods such as scType will steadily improve. The current results using older ST platforms demonstrate that scType already holds significant potential to support pathological annotations or label ST data where cell atlas references are not available. Based on our performance testing, out of the current ST assay platforms, Slide-seq seems to offer the best option for marker-based annotation methods, but this will likely change as all assay platforms strive for achieving subcellular resolution. As a conclusion, we foresee marker-based methods becoming the gold-standard in the near future when ST assay resolution and profiled number of genes keep improving further.

## Supplementary Material

btae426_Supplementary_Data

## Data Availability

The reported results can be reproduced using the data, instructions and R codes available at GitHub (https://github.com/kris-nader/sp-type). Python codes and tutorials are available https://github.com/kris-nader/sc-type-py/ and https://github.com/kris-nader/sc-type-py/blob/main/spatial_tutorial.md.
