# Supplementary material for: ScType enables fast and accurate cell type identification from spatial transcriptomics data
Source: Bioinformatics. 2024 Jun 27;40(7):btae426. doi: 10.1093/bioinformatics/btae426 (PMC11236089; doi:10.1093/bioinformatics/btae426)
Supplement: btae426_Supplementary_Data [file btae426_supplementary_data.pdf]

Supplementary Information for

ScType enables fast and accurate cell type identification from spatial transcriptomics data

Kristen Nader[1,2], Misra Tasci[3], Aleksandr Ianevski[1,2], Andrew Erickson[2,4], Emmy W. Verschuren[1], Tero Aittokallio[1,2,5,6], Mitro Miihkinen[1,2*]

Supplementary Information

- **Supplementary Table 1 | Data sets used in the study**
- **Supplementary Table 2 | Modified ground truth dataset annotations**
- **Supplementary Table 3 | Modified Reference dataset annotations before and after unifying cell labels and removing small cell populations**
- **Supplementary Figure 1 | Comparison of scType cell type annotations and pathologist's annotations in the human breast cancer data set**
- **Supplementary Figure 2 | Performance testing of scType-spatial using simulated data**
- **Supplementary Figure 3 | Comparison of different annotation tools when applied to breast cancer DCIS dataset**

Supplementary Table 1 | Data sets used in the study

Tissue sample	Spatial transcriptomic technology	Resolution (spot center-to-center)	Number of genes profiled	Number of spots	Spatial data source	Source of marker gene data
Mouse hippocampus (Fig. 1B)	Slide-seq v2	10 µm	23,264	53,173	Sensitive spatial genome wide expression profiling at cellular resolution [1]	Molecular diversity and specializations among the cells of the adult mouse brain [2]
Mouse anterior sagittal brain (Fig. 1C)	10X Genomics Visium v1	100 µm	31,053	2,696	10X Genomics datasets [3]	scTypeDB [4] Adult mouse cortical cell taxonomy revealed by single cell transcriptomics[supplement [6]
Mouse posterior sagittal brain (Fig. 1C)	10X Genomics Visium v1	100 µm	31,053	3,353	10X Genomics datasets [3]	scTypeDB [4]
Human breast cancer (Fig. 1D) (SFig. 1A)	10X Genomics Visium	100 µm	18,536	4,980	High resolution mapping of the tumor microenvironment using integrated single-cell, spatial and in situ analysis [5][*]	scFFPE [5][*]
Human breast cancer (Fig. 1D) (SFig. 1B)	10X Genomics Xenium	Not applicable - subcellular resolution.	313	166,363	High resolution mapping of the tumor microenvironment using integrated single-cell, spatial and in situ analysis [5][*]	scFFPE [5][*]

*:https://www.10xgenomics.com/products/xenium-in-situ/preview-dataset-human-breast
1:https://www.nature.com/articles/s41587-020-0739-1
2:https://linkinghub.elsevier.com/retrieve/pii/S0092-8674(18)30955-3
3:https://www.10xgenomics.com/datasets?menu%5Bproducts.name%5D=Spatial%20Gene%20Expression&query=mouse%20anterior&page=1&configure%5BhitsPerPage%5D=50&configure%5BmaxValuesPerFacet%5D=1000
4:https://www.nature.com/articles/s41467-022-28803-w
5:https://www.nature.com/articles/s41467-023-43458-x#Sec10

6: https://www.nature.com/articles/nn.4216

<table>
<tr><th>Old Annotations</th><th>New Annotations</th></tr>
<tr><td>Stromal/Endothelial</td><td rowspan="4">Stromal/Endothelial</td></tr>
<tr><td>Stromal/Endothelial/Immune</td></tr>
<tr><td>Stromal</td></tr>
<tr><td>Adipocytes</td></tr>
<tr><td>DCIS #1</td><td>DCIS #1</td></tr>
<tr><td>DCIS #2</td><td>DCIS #2</td></tr>
<tr><td>Mixed/Invasive</td><td rowspan="2">Invasive</td></tr>
<tr><td>Invasive</td></tr>
<tr><td>Myoepithelial/stromal/immune</td><td>Myoepithelial</td></tr>
<tr><td>Immune</td><td>Immune</td></tr>
<tr><td>Mixed</td><td>-</td></tr>
</table>

Supplementary Table 2 | Modified ground truth dataset annotations
To correct potential biases caused by uneven cell type taxonomies when analysing Visium data from DCIS tissue, pathology annotations were combined into 6 equally broad cell type classes.

Supplementary Table 3 | Modified Reference dataset annotations before and after unifying cell labels and removing small cell populations

Old Annotation	# of cells before	New Annotation	# of cells after
CD4+ T Cells	2899	T Cells	4742
CD8+ T Cells	1843		
DCIS 1	1862	DCIS	9909
DCIS 2	2159		
Invasive Tumor	4559		
Prolif Invasive Tumor	1329		
Macrophages 1	2964	Macrophages	3724
Macrophages 2	760		
Myoepi ACTA2+	1235	Myoepithelial	1839
Myoepi KRT15+	604		
Perivascular-like	285	-	0
IRF7+ DCs	210	-	0
LAMP3+ DCs	103		
T Cell & Tumor Hybrid	1003	-	0
NA	2834	-	0
Mast Cells	92	-	0
Stromal & T Cell Hybrid	426	-	0

A single-cell dataset from breast cancer DCIS tissue (Janesick *et al.* 2023) was randomly split into 2 sets with approximately the same proportion of each cell type. The columns describe the reference dataset annotations before and after unifying cell type labels (marked in green) and after removing cell types with insufficient number of cells (marked in red).

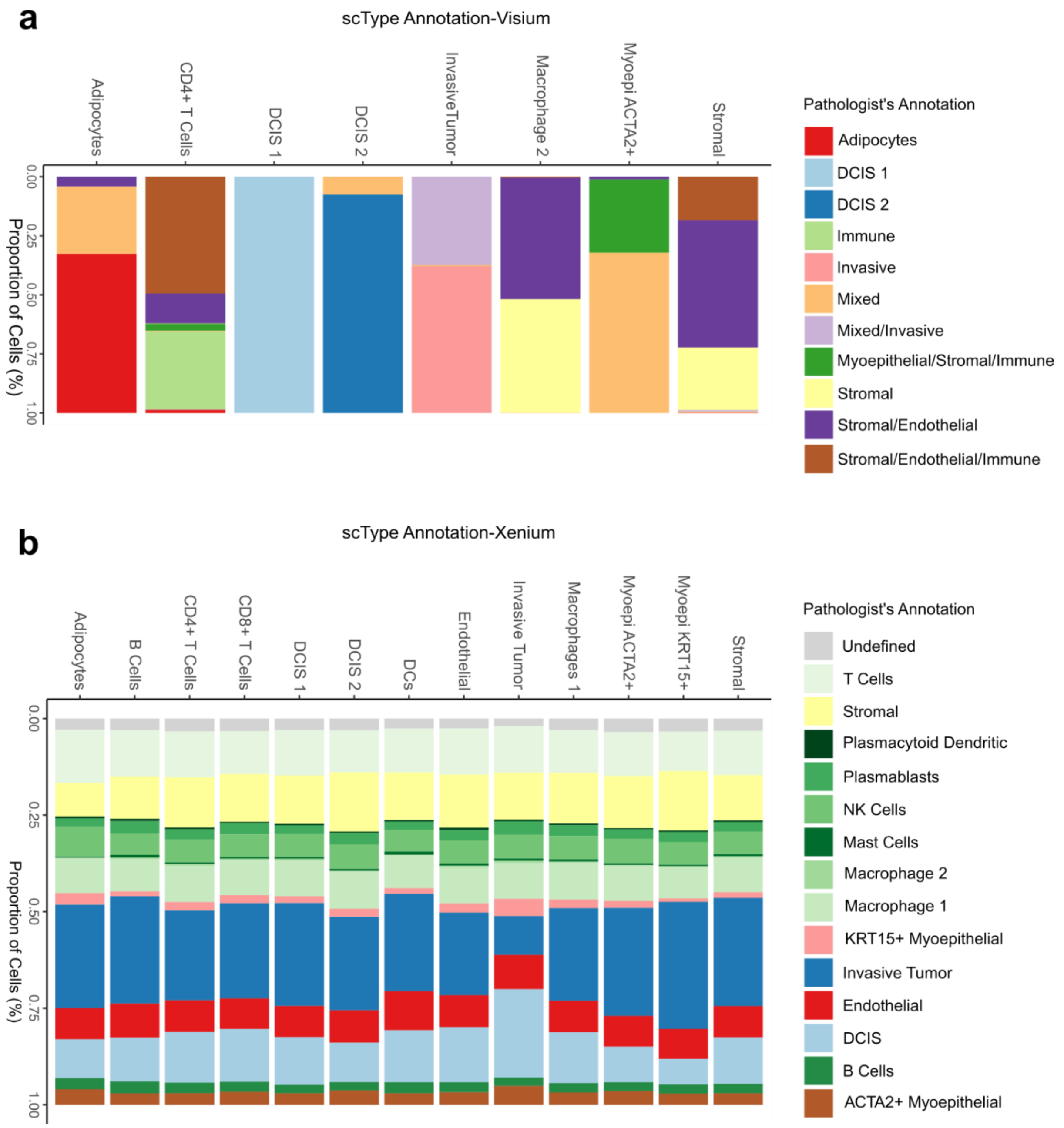

Supplementary Figure 1 | Comparison of scType cell type annotations and the pathologist's annotations in human breast cancer dataset. (**a**) scType cell type annotation accuracy in Visium data. Vertical axis shows the ground truth cell type label based on a pathologist's annotation, and horizontal axis the predicted cell type annotation. (**b**) scType cell type annotation accuracy in Xenium data. Vertical axis shows the ground truth cell type label based on a pathologist's annotation, and horizontal axis is the predicted cell type annotation.

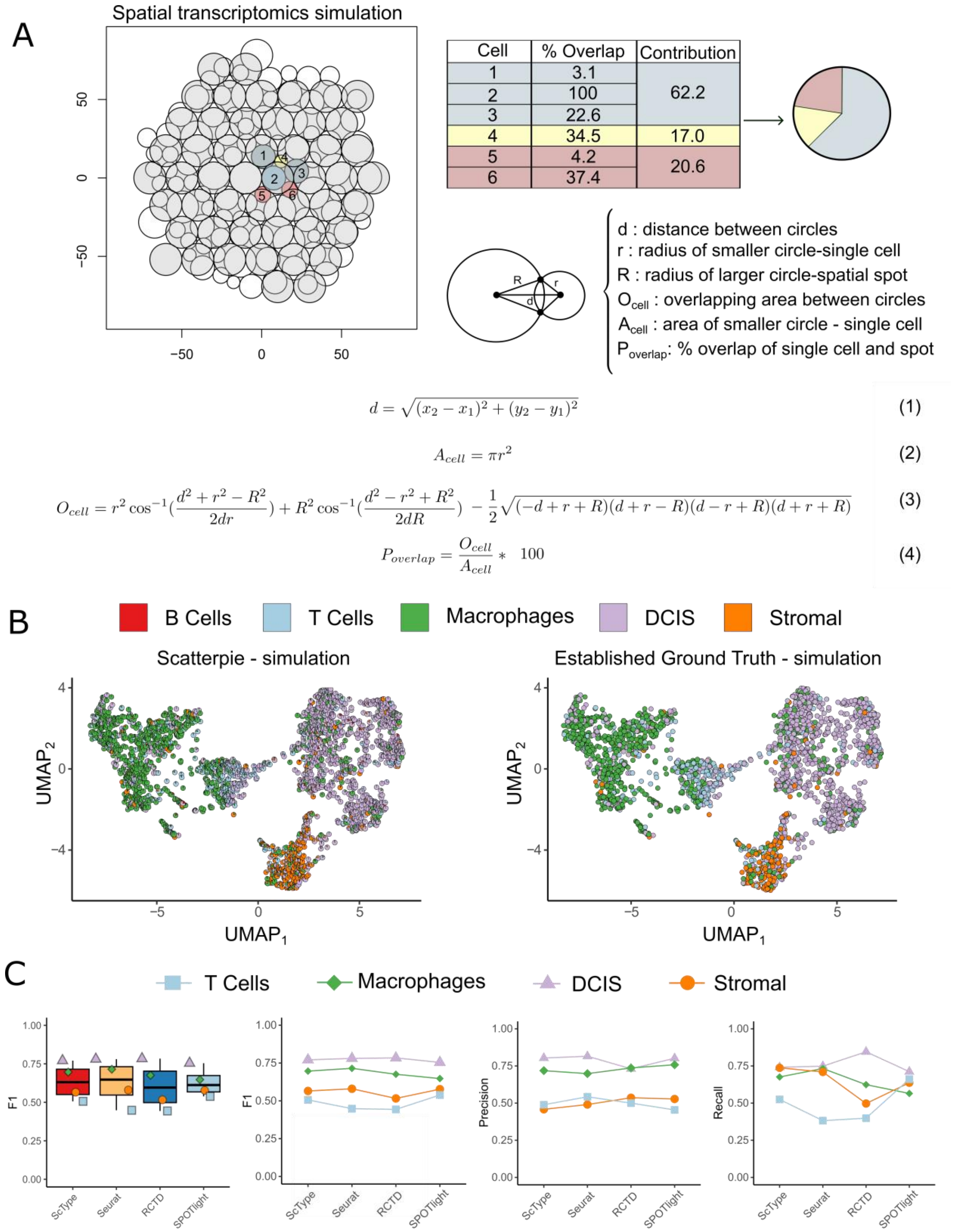

Supplementary Figure 2 | Performance testing of scType-spatial using simulated data. A) Randomly drawn single cells with cell type specific sizes were distributed into 2D grid overlaid with 10 um transcriptomics spots. The table shows the % overlap with 6 of these cells with their overlaid transcriptomic spots together with a geometric interpretation of equations 1-4 how the overlaps were calculated. The equations have been previously described by Jimenez et al. in 2016. B) The results of the simulation

displaying proportion of each cell type in each spot (left). Ground truth of the simulation using the majority cell type as annotation (right) C) Cell typing results from the simulated spatial transcriptomics data reporting F1, Precision and Recall scores.

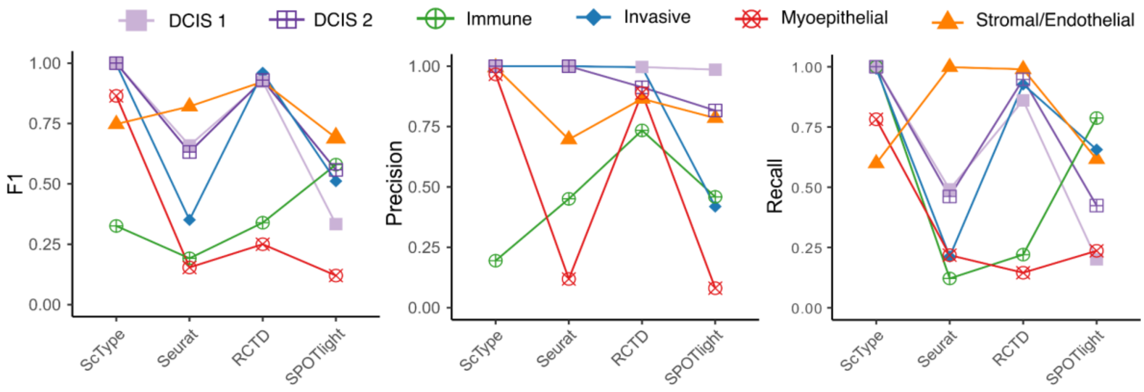

Supplementary Figure 3: Comparison of different annotation tools when applied to breast cancer DCIS dataset. A) The pathology annotations were uniformed to equal in their depth of cell typing taxonomy. The performance of both marker-based and deconvolutional tools were then assessed for each of the cell types separately. Showing are F1, precision and recall scores.

References

Janesick A, Shelansky R, Gottscho AD, Wagner F, Williams SR, Rouault M, Beliakoff G, Morrison CA, Oliveira MF, Sicherman JT, Kohlway A, Abousoud J, Drennon TY, Mohabbat SH; 10x Development Teams; Taylor SEB. High resolution mapping of the tumor microenvironment using integrated single-cell, spatial and in situ analysis. *Nat Commun*. 2023, 19;14(1):8353.

Jiménez, J., Gómez, Á., Buhrmester, M. D., Vázquez, A., Whitehouse, H., & Swann, W. B. The dynamic identity fusion index: A new continuous measure of identity fusion for web-based questionnaires. *Social Science Computer Review. 2016, 34*(2), 215–228.
